# Thrombocytopenia Is Associated with Acute Respiratory Distress Syndrome Mortality: An International Study

**DOI:** 10.1371/journal.pone.0094124

**Published:** 2014-04-14

**Authors:** Tiehua Wang, Zhuang Liu, Zhaoxi Wang, Meili Duan, Gang Li, Shupeng Wang, Wenxiong Li, Zhaozhong Zhu, Yongyue Wei, David C. Christiani, Ang Li, Xi Zhu

**Affiliations:** 1 Peking University Third Hospital, Beijing, China; 2 Beijing Friendship Hospital Affiliated to Capital Medical University, Beijing, China; 3 China-Japan Friendship Hospital, Beijing, China; 4 Beijing Chao-Yang Hospital Affiliated to Capital Medical University, Beijing, China; 5 Harvard School of Public Health, Boston, Massachusetts, United States of America; UT MD Anderson Cancer Center, United States of America

## Abstract

**Background:**

Early detection of the Acute Respiratory Distress Syndrome (ARDS) has the potential to improvethe prognosis of critically ill patients admitted to the intensive care unit (ICU). However, no reliable biomarkers are currently available for accurate early detection of ARDS in patients with predisposing conditions.

**Objectives:**

This study examined risk factors and biomarkers for ARDS development and mortality in two prospective cohort studies.

**Methods:**

We examined clinical risk factors for ARDS in a cohort of 178 patients in Beijing, China who were admitted to the ICU and were at high risk for ARDS. Identified biomarkers were then replicated in a second cohort of1,878 patients in Boston, USA.

**Results:**

Of 178 patients recruited from participating hospitals in Beijing, 75 developed ARDS. After multivariate adjustment, sepsis (odds ratio [OR]:5.58, 95% CI: 1.70–18.3), pulmonary injury (OR: 3.22; 95% CI: 1.60–6.47), and thrombocytopenia, defined as platelet count <80×10^3^/µL, (OR: 2.67; 95% CI: 1.27–5.62)were significantly associated with increased risk of developing ARDS. Thrombocytopenia was also associated with increased mortality in patients who developed ARDS (adjusted hazard ratio [AHR]: 1.38, 95% CI: 1.07–1.57) but not in those who did not develop ARDS(AHR: 1.25, 95% CI: 0.96–1.62). The presence of both thrombocytopenia and ARDS substantially increased 60-daymortality. Sensitivity analyses showed that a platelet count of <100×10^3^/µLin combination with ARDS provide the highest prognostic value for mortality. These associations were replicated in the cohort of US patients.

**Conclusions:**

This study of ICU patients in both China and US showed that thrombocytopenia is associated with an increased risk of ARDS and platelet count in combination with ARDS had a high predictive value for patient mortality.

## Introduction

Acute respiratory distress syndrome (ARDS), the most severe form of acute lung injury (ALI), is caused by several direct and indirect insults to the lung,Life threatening and often lethal. ARDS usually requires mechanical ventilation and admission to an intensive care unit (ICU); ARDS is a major cause of ICU morbidity and mortality worldwide [1]. Emerging viral diseases such as severe acute respiratory syndrome (SARS) coronavirus, H5N1 avian-origin influenza virus, and H1N1 swine-origin influenza virus not only possess the potential for pandemic spread, but also cause ARDS [2]–[4].These factors highlight the need for additional research to improve understanding of the pathogenesis of ARDS, with the ultimate goal of developing specific treatment [5].

ARDS is associated with several clinical disorders, including direct pulmonary injury from pneumonia and aspiration and extra-pulmonary injury from sepsis, trauma, and multiple transfusions [6]. Although low tidal volume ventilation, neuromuscular blockers and prone positioning ventilation have advanced treatments [7]–[9], there are currently no reliable predictive markers for early detection of ARDS in predisposed individuals. Nonetheless, many efforts have been mounted to identify biologic markers, or biomarkers, for ARDS in critically ill patients, including studies of pulmonary edema fluid, blood, and urine [10]–[12]. Recent advances on the pathophysiological mechanisms underlying ARDS have identified several clinical biomarkers to assess disease severity and outcome, including specific cytokines and their receptors (IL-6, IL-8, soluble tumor factor receptors I and II), products of epithelial and endothelial injury [receptor for advanced glycation end-products (RAGE), surfactant protein D, ICAM-1, and von Willebrand factor antigen], and markers of altered coagulation (protein C and plasminogen activator inhibitor-1) [13]. However, no individual biomarker is strongly associated with outcomes and thus cannot provide sufficient discriminating power for either diagnosis or prognosis.

Biomarker discovery and validation requires patient samples and must be combined with comprehensive clinical data collected from properly designed trials in different populations. Given the acute onset and rapid clinical progress of ARDS, a prospectively enrolled cohort study in multicenter ICUs is suitable for more complete and unbiased ARDS/ALI research [14]. Using a protocol modified from a molecular epidemiology ARDS study established in Boston, MA (Boston cohort) [14], we established a multicenter ARDS cohort in Beijing, China (Beijing cohort) in 2009. The overarching objectives of establishing this prospective cohort are to validate relevant biomarkers to ARDS, as well as genetic polymorphisms, discovered in previous USA studies in Chinese population, and discover new biomarkers of ARDS with a comprehensive sampling protocol. In this report, we present initial results on the clinical factors associated with ARDS development and mortality in individuals with or at risk for ARDS. Associated clinical factors were replicatedin the Boston cohort.

## Methods

This study was approved by the institutional review boards(IRBs) of the Peking university third hospital, Beijing Friendship Hospital, China-Japan Friendship Hospital, Beijing Chao-Yang Hospital, and Harvard School of Public Health and a written informed consent was obtained from each subject or an appropriateproxy of the patient.

### Study population

Four medical and surgical ICUs within four tertiary hospitals participated in the study; hospitals covered the metropolitan area of Beijing, China and included Peking University Third Hospital in the northwest (16 beds), Beijing Friendship Hospital in the south (16 beds), Beijing Chao-Yang Hospital in the east (14 beds), and China-Japan Friendship Hospital in the northeast (10 beds).

As an international collaboration, we used a modified study protocol for recruitment as previously described [14]. Briefly, we screened each ICU admission for eligible subjects, which were defined as critically ill patients with at least one predisposing condition for ARDS: 1) sepsis; 2) septic shock; 3) trauma; 4) pneumonia; 5) aspiration; 6) massive transfusion of packed red blood cells (PRBC; defined as >8 PRBC units during the 24 hours prior to admission); or 7) severe pancreatitis. To avoid interference in biomarker research from certain clinical conditions, exclusion criteria included:1) age <18 years; 2) history of chronic lung diseases, such as interstitial pulmonary fibrosis or bronchiolitis; 3) history of pneumonectomy; 4) treatment with immunomodulating therapy other than corticosteroids, such as granulocyte colony stimulating factor, cyclophosphamide, cyclosporine, interferon, or TNF-α antagonists; 5) presence of other immunodeficient conditions, such as HIV infection, leukemia, or neutropenia (absolute neutrophil count <1000/µl); 6) history of solid or bone marrow transplant other than autologous bone marrow transplant; and 7) directive to withhold intubation. Sepsis and septic shock were defined by the American College of Chest Physicians/Society of Critical Care Medicine (ACCP/SCCM) Consensus Conference [15].

After enrollment, subjects were followed daily for the development of ARDS, as defined by the American-European Consensus Committee (AECC) as follows [16]: a) evidence of hypoxemia with Pao_2_/Fio_2_≤200 mm Hg; b) evidence of bilateral infiltrates on chest radiographs; and c) absence of left atrial hypertension with pulmonary arterial occlusion pressure ≤18 mm Hg or no congestive heart failure. Controls were identified as at-risk patients who did not meet criteria for ARDS during the ICU stay and had no prior history of ARDS. Infiltrates on chest radiographs were defined as opacities that could not be explained completely by pleural effusions, mass, body habitus, or collapse. Upper zone redistribution and pulmonary vascular congestion were not considered infiltrates. Two pulmonary and critical care physicians interpreted daily chest radiographs; any disagreement went to a third intensivist for arbitration. All physicians underwent a consensus training session on the radiologic criteria for ARDS. All were blinded to the clinical status of the patients.

### Data and sample collection

We collected clinical data by chart review, including demographic information of age, gender, race, height, weight and medical history of ARDS, diabetes, tobacco and alcohol abuse, and liver disease. Baseline clinical information, worst vital signs, and laboratory testing results in the first 24 hours of ICU admission were collected for calculation of the Acute Physiological and Chronic Health Evaluation (APACHE II) score for severity of illness [17]. We also collected ventilatory parameters including the requirement and mode of mechanical ventilation, PaO_2_/FiO_2_ ratio, positive end-expiratory pressure, tidal volume, and peak and plateau pressures. All enrolled patients were followed until one of the following situations occurred: hospital discharge, death, or 60 days after study entry. Starting in late 2006, based on finding from ARDS network trials, Chinese ICUs universally adopted lower tidal volume for mechanical ventilation [18].

### Statistical analysis

Baseline characteristics were compared between groups with Fisher's exact test or chi-square test for dichotomous or categorical variables and with Student's *t* test or Mann–Whitney test for continuous variables. For risk analysis of ARDS development, we initially used a logistic regression model with a backward stepwise elimination algorithm to select clinical risks or predictors from the univariate analyses with *p*<0.1; the final logistic regression models also included predictors from backward elimination. For mortality analysis, we used the log-rank test as a univariate measure of association and employed Cox proportional hazards models to investigate each clinical variable's effect on clinical outcome. We used the time-dependent receiver operating characteristic (ROC) method to determine the best cut-off value of thrombocytopenia in the prediction of prognosis of critically ill patients by exploring the area-under-the-curve (AUC) values at 60-day mortality, and selected the maximal sumof sensitivity and specificity [19]. All analyses were performed with the SAS statistical software package (version 9.31, SAS Inc., Cary, NC), and *p*<0.05 was considered statistically significant.

## Results

Between July 2010 and April 2012, we recruited 178 patients with at least one predisposing condition for ARDS from the participating hospitals in Beijing, China. The majority of patients were male (n = 125, 70%) and the mean age was 63 years (median: 69; interquartile range: 51–78 years) (Table 1). Median APACHE II score was 16 (interquartile range: 12–22) and median length of time in the ICU was 10.5 days (interquartile range: 5–17 days). Mechanical ventilation was used on 149 patients (84%) for a median length of six days (interquartile range: 2–11 days). Thirty-nine patients had diabetes, and one patient had chronic kidney disease.

**Table 1:** Baseline characteristics of the Beijing cohort.

	Non-ARDS (n = 103)	ARDS (n = 75)	*p*
**Female, n (%)**	34 (33.0)	19(25.3)	0.27
**Age, median (range)**	70(18–99)	67 (18–91)	0.32
**Smoking history, n (%)**	21 (20.4)	20 (26.7)	0.34
**Baseline severity of illness (1^st^ 24 hours of ICU admission)**			
APACHE II, median(range)*	15 (4–39)	17 (6–35)	0.79
Systolic BP,<90 mmHg, n (%)	33 (32.0)	21 (28)	0.64
Heart rate,>100 beats/min, n (%)	82 (79.6)	57 (76)	0.84
Respiratory rate,>30 breaths/min, n (%)	27 (26.7)	31 (43.1)	0.03
Creatinine,>2.0 mg/L, n (%)	37 (35.9)	22 (29.3)	0.36
Bilirubin,>2.0 mg/dL, n (%)	35 (34.0)	21 (28)	0.23
Thrombocytopenia, ≤80×10^9^ platelets/L, n (%)	26 (25.2)	28 (37.3)	0.07
Albumin,<25 g/dL, n (%)	25 (61)	16 (39)	0.57
Arterial pH,<7.33, n (%)	35 (35)	28 (37.3)	0.74
Arterial pH,<7.22, n (%)	4 (3.9)	7 (9.3)	0.14
**Comorbidities, n (%)**			
Diabetes	23 (22.3)	16 (21.3)	0.87
**Predisposing conditions for ARDS, n (%)**			
Sepsis syndrome	80 (77.7)	69 (92)	0.01
Septic shock	47 (45.6)	31 (41.3)	0.57
Pneumonia	35 (34)	29 (38.7)	0.52
Pancreatitis	11 (10.7)	12 (16)	0.30
Trauma	3 (2.9)	3 (4)	0.70
Multiple transfusions	10 (9.7)	5 (6.7)	0.47
Aspiration	2 (1.9)	10 (13.3)	0.004
>1 risk for ARDS	38 (36.9)	51 (68)	<0.0001
Direct pulmonary injury vs. external pulmonary injury†	36 (35)	39 (52)	0.02
**Clinical outcomes**			
60-day mortality, n (%)	38 (36.9)	31 (41.3)	0.55
Days in ICU, median (IQR)	8 (5–14)	11 (7–27)	0.21
Days on mechanical ventilation, median (IQR)	5 (2–10)	7 (3–14)	0.53

ARDS = acute respiratory distress syndrome; APACHE = Acute Physiology and Chronic Health Evaluation; ICU = intensive care unit; IQR = interquartile range.

*APACHE II score was calculated with all components within 24 hours of ICU admission.

† Pneumonia, aspiration, pulmonary contusions, or sepsis from lower pulmonary source were categorized as direct pulmonary injury; sepsis from an extrapulmonary source, trauma without pulmonary contusions, and multiple transfusions were categorized as external pulmonary lung injury. Patients with both direct and external pulmonary injuries were considered to have direct lung injury.

### ARDS development

During hospitalization, 75 (41%) patients developed ARDS;among identified cases,31 (41%) and 70 (93%) of the 75 ARDS patients were diagnosed within the first 24 and 72 hours of ICU admission, respectively. There were no significant differences in age, gender, smoking status, and initial APACHE II score between ARDS patients and at-risk non-ARDS patients (Table 1). In addition, the major physiological variables during the first 24 hours of ICU admission were comparable, except that patients who developed ARDS had higher respiratory rates (*p* = 0.025). Although not significant, ARDS patients were in the ICU longer (ARDS median = 13 days; non-ARDS median = 9 days; *p* = 0.21) and on mechanical ventilation longer (ARDS median = 7 days; non-ARDS median = 5 days;*p* = 0.53) than non-ARDS patients. Low tidal volume (∼7 ml/kg) was used in treating patients with mechanical ventilation. Although patient specific data was not available, protocolled low tidal volume ventilation was standardized in study ICUs.

Among predisposing conditions for ARDS in all enrolled patients, sepsis and/or septic shock (n = 149, 83%) were the most common, followed by pneumonia (n = 64, 36%) and severe acute pancreatitis (n = 23, 13%). Table 1 also shows the proportions of patients who developed ARDS with each condition. Patients with sepsis (*p* = 0.01), aspiration (*p* = 0.004), or multiple predisposing conditions (*p*<0.0001) had significantly higher ARDS risk. Patients with direct pulmonary injury were more likely to develop ARDS than patients with extra pulmonary injury (*p* = 0.02). From multivariate modeling, sepsis [odds ratio (OR) = 5.58; 95% confidence intervals (CI) = 1.70–18.26; *p* = 0.005), direct pulmonary injury (OR = 3.22; 95% CI = 1.60–6.47; *p* = 0.001), and thrombocytopenia (platelet count <80×10^3^/µL) (OR = 2.67; 95% CI = 1.27–5.62; *p* = 0.005) [20] were associated with development of ARDS.Respiratory rate (>30 breaths/min), aspiration, and >1 risks for ARDS were also evaluated in model selection but were eliminated during model selection (not significant). APACHE II score (removing age and gender components), age, and gender were forced in as covariatesbut not significant in logistic regression analyses. Known factors related to ARDS, including septic shock, diabetes, and alcohol use, were also tested either by forcing as covariates, individually or combined into the model, and did not change the significant associations of sepsis, direct pulmonary injury, and thrombocytopenia with the development of ARDS (data not shown).Because drinking habits in China differ from those in the U.S.,and it was difficult to develop a comparable criterion for alcohol abuse, we did not including alcohol abuse as risk factor in the analysis.

We further conducted a stratified analysis and found that thrombocytopenia was significantly associated with ARDS in both the Beijing cohort (univariate analysis, *p* = 0.01) and the Boston cohort (univariate analysis, *p*<0.0001) (Table S1), which has 851 ARDS and 1,027 non-ARDS patients recruited at Massachusetts General Hospital in Boston, USA [21], in the subgroup patients with septic shock, but not in non-septic shock subgroup (*p* = 0.95 and *p* = 0.15, respectively).

Some patients had already developed ARDS before ICU admission, and this subgroup caseswas usually mixed with those patients who were diagnosed ARDS during the first 24 hours of ICU admission, together accounting for a total of 41% ARDS in the Beijing cohort and 40% in the Boston cohort (340 of 851 identified cases). Since the thrombocytopenia was defined by the lowest platelet counts during the first 24 hours of ICU admission in these cohorts, some patients developed thrombocytopenia before the onset of ARDS, who were difficult to be distinguished within this subgroup ARDS, and could interfere with the finding that thrombocytopenia was associated with development of ARDS. We then performed a nested analysis on a clean subgroup patients, by removing ARDS patients who were diagnosed during the first 24 hours of ICU admission, and found that thrombocytopenia was still significantly associated with ARDS risk (OR = 4.04; 95% CI = 1.41–11.59; *p* = 0.009). Because of the small sample size of the Beijing cohort, we further conducted the sensitivity test in the Boston cohort in 411 ARDS cases and 1,027 non-ARDS patients after removing ARDS patients who were diagnosed during the first 24 hours of ICU admission. Thrombocytopenia were significantly associated with development of ARDS (OR = 1.85; 95% CI = 1.33–2.58; *p* = 0.0003), with the adjustment of APACHE II score (removing age and gender components), age, gender, sepsis, trauma, blood transfusion, direct pulmonary injury, and alcohol use, which was consistent with the results of entire sample set (OR = 1.71; 95% CI = 1.27–2.31; *p* = 0.0005).

### Mortality

The 60-day mortality rate for all patients was 39%, and the development of ARDS did not increase mortality risk (Table 1). Among predisposing conditions for ARDS, septic shock was associated with increased mortality (*p* = 0.014), but pancreatitis was associated with decreased mortality (*p* = 0.01) in ARDS patients (Table 2). In contrast, pneumonia (*p* = 0.002) and external pulmonary injury (*p* = 0.013) had higher mortality rates in non-ARDS patients.

**Table 2:** Risk factors for 60-day mortality.

		ARDS				Non-ARDS				All
		Total	Death	75% survival	*p*	Total	Death	75% survival	*p*	*p*
		n	n (%)	Day		n	n (%)	Day		
Gender					0.362				0.945	0.461
	Male	56	25 (45)	29		69	25 (36)	38		
	Female	19	6 (32)	17		34	13 (38)	14		
Age					0.053				0.023	0.004
Smoking status					0.745				0.24	0.321
	Non-smoker	55	23 (42)	27		81	32 (40)	25		
	Smoker	20	8 (40)	17		21	5 (24)	-		
Diabetes					0.707				0.988	0.717
	Non-diabetic	59	22 (37)	20		80	29 (36)	17		
	Diabetic	16	9 (56)	39		23	9 (39)	28		
**Baseline severity of illness (1st 24 hours of ICU admission)**										
APACHE II*					0.048				0.0002	<0.0001
Systolic blood pressure					0.364				0.527	0.951
	≥90 mmHg	52	20 (38)	29		70	27 (39)	25		
	<90 mmHg	21	10 (48)	11		33	11 (33)	19		
Heart rate					0.278				0.093	0.55
	≤100 beats/min	15	8 (53)	31		20	4 (20)	57		
	>100 beats/min	57	22 (39)	17		82	34 (41)	17		
Respiratory rate					0.403				0.019	0.23
	≤30 breaths/min	41	18 (44)	20		74	23 (31)	45		
	>30 breaths/min	31	12 (39)	29		27	15 (56)	11		
Creatinine					0.997				0.004	0.028
	≤2.0 mg/L	53	22 (42)	20		66	18 (27)	60		
	>2.0 mg/L	22	9 (41)	27		37	20 (54)	12		
Bilirubin					0.895				0.296	0.45
	≤2.0 mg/dL	53	23 (43)	24		59	20 (34)	42		
	>2.0 mg/dL	21	8 (38)	11		35	14 (10)	11		
Thrombocytopenia					0.022				0.436	0.024
	Platelet >80×10^9^/L	46	15 (33)	34		77	27 (35)	19		
	Platelet ≤80×10^9^/L	28	16 (57)	9		26	11 (42)	21		
Albumin					0.59				0.071	0.297
	≥25 g/dL	59	22 (37)	20		75	31 (41)	18		
	<25 g/dL	16	9 (56)	25		25	6 (24)	-		
Arterial pH					0.208				0.149	0.051
	≥7.33	47	16 (34)	31		67	21 (31)	42		
	<7.33	28	15 (54)	13		36	17 (47)	12		
**Predisposing conditions for ARDS**										
Sepsis					0.959				0.831	0.959
	No sepsis	6	2 (33)	19		23	7 (30)	17		
	Sepsis	69	29 (42)	24		80	31 (39)	22		
Septic shock					0.014				0.273	0.486
	No septic shock	44	13 (29)	34		56	22 (39)	17		
	Septic shock	31	18 (58)	10		47	16 (34)	28		
Pneumonia					0.382				0.001	0.002
	No pneumonia	46	17 (37)	19		68	19 (28)	42		
	Pneumonia	29	14 (48)	24		35	19 (54)	12		
Pancreatitis					0.01				0.967	0.061
	No pancreatitis	63	30 (48)	17		92	34 (37)	25		
	Pancreatitis	12	1 (8)	-		11	4 (36)	8		
Trauma					0.163				0.241	0.071
	No trauma	72	31 (43)	20		100	38 (38)	19		
	Trauma	3	0 (0)	-		3	0 (0)	-		
Multiple transfusions					0.629				0.365	0.311
	No multiple transfusions	70	29 (41)	24		93	36 (39)	19		
	Multiple transfusions	5	2 (40)	46		10	2 (20)	-		
Aspiration					0.92				0.434	0.856
	No aspiration	65	27 (42)	24		101	38 (38)	19		
	Aspiration	10	4 (40)	7		2	0 (0)	-		
Pulmonary injury†					0.812				0.003	0.013
	Direct pulmonary injury	36	14 (39)	17		67	19 (28)	42		
	External pulmonary injury	39	17 (44)	29		36	19 (53)	12		

ARDS = acute respiratory distress syndrome; APACHE = Acute Physiology and Chronic Health Evaluation; ICU = intensive care unit.

*APACHE II score was calculated with all components within 24 hours of ICU admission;

† Pneumonia, aspiration, pulmonary contusions, or sepsis from lower pulmonary source were categorized as direct pulmonary injury; sepsis from an extrapulmonary source, trauma without pulmonary contusions, and multiple transfusions were categorized as external pulmonary lung injury. Patients with both direct and external pulmonary injuries were considered to have direct lung injury.

Univariate examination of demographic characteristics and physiologic variables in the first 24 hours of ICU admission revealed that higher APACHE II scores and older age were associated with increased mortality for both ARDS and non-ARDS patients (Table 2). Thrombocytopenia was significantly associated with mortalityof ARDS (*p* = 0.022) but not non-ARDS (*p* = 0.436) patients. In contrast, high serum creatinine levels (>2.0 mg/L) were associated with higher mortality in non-ARDS (*p* = 0.004) but not ARDS (*p* = 0.997) patients. There were no statistically significant differences between survivors and non-survivors for gender, history of diabetes, and tobacco or alcohol use.

In multivariate analysis, APACHE II score was consistently associated with increased mortality in ARDS, non-ARDS, and all patients (Table 3). Thrombocytopenia was a mortality covariate for ARDS and all patients, but not for non-ARDS patients (Table 3). When replaced with coagulation points of the Sequential Organ Failure Assessment score (SOFA), thrombocytopenia remained associated with higher mortality in ARDS [adjusted hazard ratio (AHR) = 1.38; 95% CI = 1.07–1.57; *p* = 0.04]and all patients (AHR = 1.30; 95% CI = 1.07–1.57; *p* = 0.008), but not in non-ARDS patients (AHR = 1.25; 95% CI = 0.96–1.62; *p* = 0.09). To replicate our findings, we analyzed data from the Boston cohort. Although univariate analyses identified more physiologic variables in the first 24 hours of ICU admission and ARDS-predisposing conditions significantly associated with mortality (Table S1 and S2), multivariate analyses identified APACHE II score and thrombocytopenia as major risk factors for mortality in ARDS, non-ARDS, and all patients (Table 3). We also found similar results when thrombocytopenia was replaced with coagulation points of the SOFA score (data not shown).

**Table 3:** Multivariable analysis of mortality predictors in Beijing and Boston cohorts.

		Beijing cohort		Boston cohort	
	Covariate	AHR (95% CI)	*p*	AHR (95% CI)	*p*
**ARDS**		N = 75		N = 851	
	APACHE II	1.05 (1.00–1.10)	0.04	1.05 (1.03–1.08)	<0.0001
	Thrombocytopenia	2.20 (1.09–4.46)	0.03	1.69 (1.23–2.32)	0.001
	Age	NS		1.03 (1.02–1.04)	<0.0001
	Bilirubin, >2.0 mg/dL	NS		1.45 (1.08–1.95)	0.01
**Non-ARDS**		N = 103		N = 1027	
	APACHE II	1.08 (1.04–1.12)	0.0002	1.07 (1.04–1.10)	<0.0001
	Thrombocytopenia	1.29 (0.64–2.59)	0.48	1.63 (1.14–2.34)	0.008
	Pneumonia	2.04 (1.02–4.09)	0.04	NS	
	Age	NS		1.03 (1.02–1.04)	<0.0001
	Aspiration	NS		1.79 (1.18–2.73)	0.006
**All patients**		N = 178		N = 1878	
	APACHE II	1.07 (1.03–1.10)	<0.0001	1.07 (1.05–1.08)	<0.0001
	Thrombocytopenia	1.89 (1.16–3.08)	0.01	1.97 (1.56–2.48)	<0.0001
	Pneumonia	2.01 (1.24–3.26)	0.005	NS	
	Age	NS		1.02 (1.02–1.03)	<0.0001
	Trauma	NS		0.09 (0.01–0.67)	0.02
	Direct pulmonary injury	NS		1.36 (1.12–1.65)	0.002

ARDS = acute respiratory distress syndrome; APACHE = Acute Physiology and Chronic Health Evaluation; AHR = adjusted hazard ratio; CI = confidence intervals; NS = not selected in multivariate modeling.

**Figure 1:**
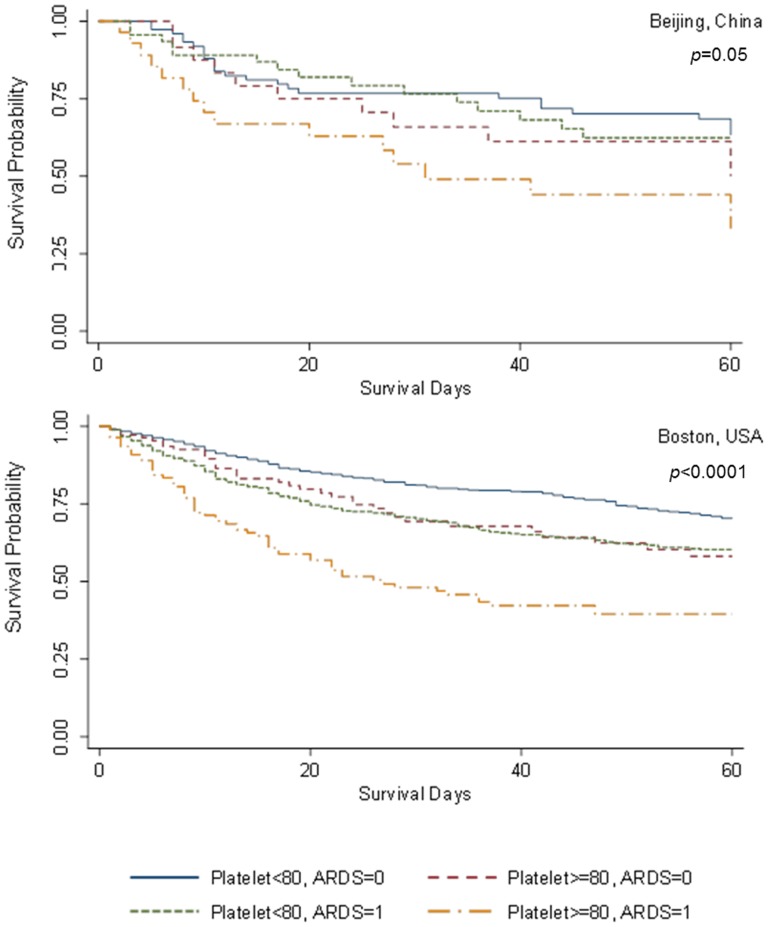
Survival curves of the effects of thrombocytopenia and acute respiratory distress syndrome (ARDS) on 60-day mortality. Univariate survival analyses show Kaplan-Meier curves of thrombocytopenia (platelet count <80×10^3^/µL) and ARDS development on 60-day mortality among critically ill patients at-risk for ARDS in the Beijing cohort (upper panel, *p* = 0.05) and the Boston cohort (lower panel, *p*<0.0001). Blue: non-thrombocytopenia & non-ARDS; red: thrombocytopenia & non-ARDS; green: non-thrombocytopenia & ARDS; orange: thrombocytopenia & ARDS.

**Figure 2:**
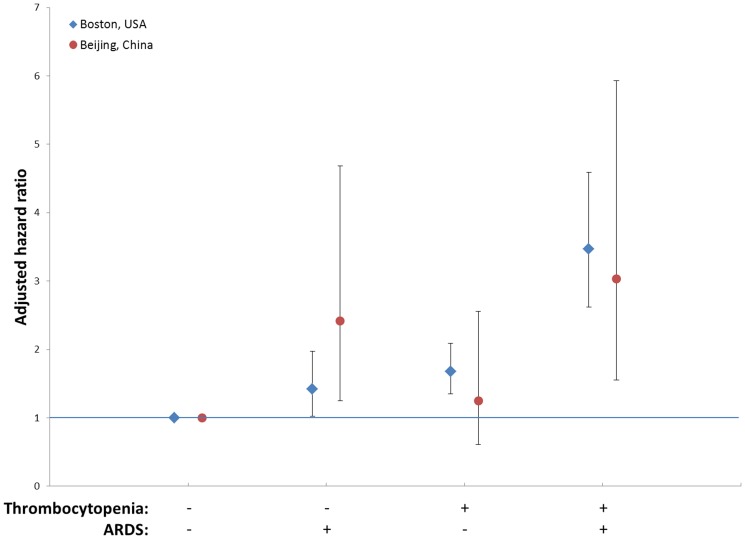
Adjusted hazard ratios of thrombocytopenia and acute respiratory distress syndrome (ARDS) on 60-day mortality. Multivariate analyses show adjusted hazard ratios (95% confidence intervals) of thrombocytopenia(platelet count <80×10^3^/µL) and ARDS development on 60-day mortality among critically ill patients at-risk for ARDS in the Beijing cohort (red) and the Boston cohort (blue), adjusted by APACHE II.

We further investigated the interaction between thrombocytopenia and ARDS on mortality of all patients by creating a combined covariate of the Boston and Beijing cohorts. In both univariate (Figure 1) and multivariate (Figure 2) analyses, the combination of thrombocytopenia and ARDS had consistently higher patient mortality.

Taking advantage of the size of the Boston cohort, we conducted a sensitivity analysis to determine the optimal platelet count for prognosis.With adjustmentsfor age, gender, APACHE II score, and sepsis, a platelet count of 104×10^3^/µLhad the maximal ROC value (AUC = 0.661; sensitivity = 0.508; specificity = 0.739; *p* = 0.0007). A sensitivity analysis confirmed a platelet count of 100×10^3^/µL by considering a series of stepped (10×10^3^/µL)cut-off values from 50×10^3^/µLto 150×10^3^/µL(Table 4).The Beijing cohort replicated this value (platelet counts<100×10^3^/µL& non-ARDS: AHR = 2.42; 95% CI = 1.25–4.68; *p* = 0.009; platelet counts≥100×10^3^/µL& ARDS: AHR = 1.25; 95% CI = 0.61–2.56; *p* = 0.551; platelet counts<100×10^3^/µL& ARDS: AHR = 3.03; 95% CI = 1.55–5.93; *p* = 0.001).

**Table 4:** Sensitivity analysis of combined effects of thrombocytopenia and ARDS†.

cutoff point for platelet count*	Platelet count <cutoff & Non-ARDS		Platelet count ≥cutoff & ARDS		Platelet count <cutoff & ARDS	
	AHR†	*p*	AHR†	*p*	AHR†	*p*
50	1.39(0.81,2.36)	0.2286	1.79(1.47,2.18)	6.50E-09	3.62(2.45,5.34)	8.80E-11
60	1.36(0.84,2.19)	0.2107	1.74(1.42,2.13)	6.30E-08	3.91(2.78,5.51)	5.40E-15
70	1.37(0.90,2.07)	0.1407	1.76(1.43,2.15)	5.60E-08	3.43(2.47,4.76)	1.80E-13
80	1.51(1.04,2.19)	0.0308	1.75(1.42,2.16)	1.50E-07	3.40(2.51,4.60)	2.10E-15
90	1.44(1.01,2.05)	0.0459	1.68(1.36,2.08)	1.90E-06	3.66(2.74,4.87)	<1E-16
**100**	**1.42(1.02,1.97)**	**0.0356**	**1.68(1.35,2.09)**	**3.00E-06**	**3.47(2.62,4.59)**	**<1E-16**
110	1.35(0.99,1.85)	0.0587	1.68(1.34,2.09)	5.20E-06	3.19(2.42,4.19)	1.10E-16
120	1.39(1.03,1.88)	0.0316	1.69(1.35,2.12)	5.00E-06	3.12(2.38,4.09)	2.20E-16
130	1.33(0.99,1.77)	0.0543	1.70(1.35,2.15)	7.20E-06	2.84(2.18,3.69)	1.20E-14
140	1.25(0.94,1.65)	0.1213	1.73(1.36,2.19)	5.80E-06	2.58(1.99,3.36)	1.60E-12
150	1.27(0.97,1.67)	0.0856	1.73(1.36,2.21)	1.00E-05	2.58(1.99,3.34)	8.80E-13

ARDS = acute respiratory distress syndrome; AHR = adjusted hazard ratio.

*Each line represents a multivariate cox regression with adjustment of age, gender, APACHII score, and sepsis; Platelet count (×10^3^/µL) was dichotomized by cutoff point;

† AHR was estimated by comparison with non-thrombocytopenia/non-ARDS group.

## Discussion

This prospective multicenter cohort was established using a modified protocol originally implemented in the Boston cohort [14]. Among at-risk ICU patients, 41% developed ARDS during ICU admission, and a majority of those patients (93%) developed ARDS within the first 72 hours of admission. These observations are consistent with previous reports in the mostly-Caucasian Boston cohort [14]. Moreover, the profiles of baseline physiologic variables and the major clinical risk factors between ARDS and at-risk non-ARDS patients are similar to previous reports from Chinese [22], [23] andAmerican [14] ICUs.Furthermore, the observation of high baseline respiratory rate (>30 breaths/min) associated with ARDS cases was consistent withthe findings from several previous studies [24]–[26].

In this cohort, in addition to sepsis and direct pulmonary injury, thrombocytopenia was associated with the development of ARDS.Enhanced platelet activation resulting in platelet deposition within the damaged pulmonary microvasculature has been supported by several clinical and preclinical studies of ALI [27], [28], and thrombocytopenia has been reported as a key feature of SARS [29]. In the Boston cohort, thrombocytopenia (named hematologic failure) was also identified as a risk factor for ARDS in multivariate analysis [14]. In another cohort of ALI in Rochester, Minnesota (Mayo Clinic), however, researchers did not observe significant difference of platelet count between ALI and non-ALI patients with septic shock [18]. Since the Rochester cohort only focuses on a subgroup ICU patients with septic shock, our stratified analysis revealed that thrombocytopenia was significantly associated with ARDS in both the Beijing cohort and the Boston cohort in the subgroup patients with septic shock, but not in non-septic shock subgroup. The different results might be explained by that the Beijing cohort and the Boston cohort focused on ARDS, which is the most severe form of ALI.

A major finding of this study is the association ofthrombocytopenia with increased ARDS mortality. Extensive evidence demonstrates that platelet count and function are independently associated with increased ICU morbidity and mortality [30]. Although thrombocytopenia is a well-established prognostic marker for mortality in patients with sepsis and septic shock [31], which are risk factors for developing ARDS, thrombocytopenia has been inconsistently associated with ARDS mortality in two previous studies with small patient series representing non-contemporary treatment eras [32], [33]. Besides APACHE II score, thrombocytopenia was the only risk factor for ARDS mortality identified in the Beijing cohort. Further, this association was replicatedwitha larger population and different ethnicities in the Boston cohort. These results provide strong evidence that thrombocytopenia is a prognostic marker for ARDS mortality.

In both Beijing and Boston cohorts, the combination of thrombocytopenia and ARDS further increased risk of 60-day mortality among critically ill patients. Thrombocytopenia in ICU patients is caused by multiple factors [34] and is considered a marker of illness severity with Multiple Organ Dysfunction Scores (MODS), SimplifiedAcute Physiology Scores (SAPS), and APACHE scores.Sepsis alone can cause moderate thrombocytopenia, as maladaptive platelet-neutrophil interactionssignificantly increase platelet activation and aggregation, as well as tissue injury [35]. The lung epithelium is central to both the pathogenesis and resolution of ARDS, and intra-alveolar coagulation changes (e.g., platelet-fibrin deposition and pulmonary vascular thrombi) are hallmarks of pathologic changes in ARDS [36]. Thus, thrombocytopenia likely contributes to the development of ARDS; in return, the coexistence of ARDS may aggravate thrombocytopenia to increase mortality of critically ill patients.

We used the same platelet count criterion from the Boston cohort (<80×10^3^/µL)to define thrombocytopenia [14]. Although platelet counts are routinely measured daily in the ICU, the epidemiology of thrombocytopenia in critically ill patients has not been well studied. Further, illness severity scoring systems inconsistently consider platelet count;for example, the SOFA score incorporates platelet count, whereas the APACHE score does not. Different platelet count thresholds have been used in epidemiological studies for the prevalence, incidence, risk factors, and consequences of thrombocytopenia among critically ill patients [37]. Currently, the RAND/UCLA Appropriateness Methodrecommends a platelet count threshold of <100×10^3^/µL, or a >30% decrease in platelet counts, for epidemiological research of thrombocytopenia [20]. Taking advantage of a large patient population in the Boston cohort, we conducted a sensitivity analysis and determined that platelet count (<100×10^3^/µL) was a significant prognostic marker for ARDS. We further replicated this cut-point value in the Beijing cohort.

There were some differences between the Beijing and Boston cohorts. When evaluated individually, there were different association profiles for thrombocytopenia and ARDS with mortality of all patients with at least one risk factor for ARDS. Thrombocytopenia was significantly associated with higher mortality in the Beijing cohort, but not the Boston cohort. Conversely, ARDSwas associated with higher mortality in the Boston cohort, but not the Beijing cohort. It is unexpected that ARDS did not increase mortality in the Beijing cohort, and. it is counter-intuitive that in the univariate analysis thrombocytopenia would be associated with increased mortality in the ARDS group but not in the non-ARDS group given that thrombocytopenia is a marker of severity of illness in critical care populations generally. However, the raw numbers were in the direction of higher mortality with lower platelets in the non-ARDS group, these findings could be explained by the limitation of multiple subgroup analysis in a relatively small dataset. Moreover, for several known risk factors or comorbidities of ARDS [14], [24]–[26], such as septic shock, pneumonia, pancreatitis, trauma, multiple transfusions, and diabetes, we did not observe significant different between ARDS and non-ARDS patients in the Beijing cohort. Due to the relative scarcityand high cost of medical resources to the general Chinese population, the participating hospital ICUs in the Beijing cohortmay have admitted more severely ill patients, resulting in less difference in illness severity between ARDS and non-ARDS patients. Accordingly, although most physiologic variables of the first 24 hours of ICU admission, including APACHE score, were comparable between ARDS and non-ARDS Beijing cohort patients, thrombocytopenia (platelet counts <80×10^3^/µL) was more common in the Beijing cohort than the Boston cohort (30.3% vs. 13.9%, respectively; *p*<0.0001). It is also possible that different ethnicities account for the observed differencesbetween cohorts.

Based on the Boston cohort, our study protocol was modified by the inclusion of severe pancreatitis as a predisposing condition for ARDS. Severe pancreatitis is a well-established risk factor for the development of ARDS [38] and is frequently observed in critically ill patients in China. In the Beijing cohort, we identified 23 (12.9%) cases of severe pancreatitis, similar to a previous report of 15.6% in a large Chinese ICU survey [39]. About 52% of severe pancreatitis cases eventually developed ARDS during ICU admission. Severe pancreatitis was not associated with ARDS risk, but was associated with lower mortality. However, the Beijing study is limited by a small sample size. With the patients' enrollment keeps, we will further evaluate severe pancreatitis as a clinically important factor in the development and outcome of ARDS.

## Conclusion

This study describes the successful establishment of a prospective, multicenter cohort study of critically ill patients at-risk for ARDS in Beijing, China. Initial characterization of the clinical factors associated with ARDS risk and mortality revealed an association between thrombocytopenia and ARDS mortality. We replicated these findings in the larger and more diverse Boston cohort, suggesting that the Beijing cohort can provide comprehensive data and samples to identify biomarkers for the early diagnosis, prognosis, and treatment of ARDS.

## Supporting Information

Table S1 **Risk factors for 60-day mortality in Boston cohort.**
(DOCX)

Table S2 **Risk factors for ARDS in Boston cohort.**
(DOCX)
