# Supplementary material for: Thrombocytopenia Is Associated with Acute Respiratory Distress Syndrome Mortality: An International Study
Source: PLoS One. 2014 Apr 14;9(4):e94124. doi: 10.1371/journal.pone.0094124 (PMC3986053; doi:10.1371/journal.pone.0094124)
Supplement: Table S1 — Risk factors for 60-day mortality in Boston cohort. (DOCX) [file pone.0094124.s001.docx]

**Supplemental Table S1. Risk factors for 60-day mortality in Boston cohort**

|  |  | | ARDS | | | | | |  | Non-ARDS | | | | All |
| --- | --- | --- | --- | --- | --- | --- | --- | --- | --- | --- | --- | --- | --- | --- |
|  |  | | Total | | | Death | 75% survival | *p* |  | Total | Death | 75% survival | *p* | *p* |
|  |  | | n | | | n (%) | day |  |  | n | n (%) | day |  |  |
| Gender |  | |  | | |  |  | 0.887 |  |  |  |  | 0.447 | 0.658 |
|  | Male | | 553 | | | 192 (35) | 19 |  |  | 659 | 141 (21) | 48 |  |  |
|  | Female | | 320 | | | 112 (35) | 17 |  |  | 451 | 98 (22) | 42 |  |  |
| Age |  | |  | | |  |  | <0.0001 |  |  |  |  | <0.0001 | <0.0001 |
| Smoking status | |  |  | | |  |  | 0.009 |  |  |  |  | 0.02 | 0.007 |
|  | Non-smoker | | 477 | | | 188 (39) | 15 |  |  | 762 | 170 (22) | 43 |  |  |
|  | Smoker | | 260 | | | 73 (28) | 28 |  |  | 238 | 34 (14) | - |  |  |
| Diabetes |  | |  | | |  |  | 0.496 |  |  |  |  | 0.489 | 0.65 |
|  | Non-diabetic | | 667 | | | 230 (34) | 19 |  |  | 774 | 160 (21) | 46 |  |  |
|  | Diabetic | | 196 | | | 73 (37) | 16 |  |  | 329 | 77 (23) | 43 |  |  |
| **Baseline severity of illness (1st 24 hours of ICU admission)** | | | | | | | | |  |  |  |  |  |  |
| APACHE II | | | | |  |  |  | <0.0001 |  |  |  |  | <0.0001 | <0.0001 |
| Systolic blood pressure | | | | | |  |  | 0.108 |  |  |  |  | 0.057 | 0.055 |
|  | ≥90 mmH | | 283 | | | 93 (33) | 26 |  |  | 277 | 47 (17) | - |  |  |
|  | <90 mmH | | 606 | | | 221 (36) | 14 |  |  | 833 | 192 (23) | 43 |  |  |
| Heart rate | | |  | | |  |  | 0.147 |  |  |  |  | 0.206 | 0.023 |
|  | ≤100 beats/min | | 182 | | | 56 (31) | 27 |  |  | 309 | 56 (18) | 49 |  |  |
|  | >100 beats/min | | 685 | | | 245 (36) | 16 |  |  | 800 | 183 (23) | 44 |  |  |
| Respiratory rate | | |  | | |  |  | 0.002 |  |  |  |  | 0.342 | 0.0002 |
|  | ≤30 breaths/min | | 479 | | | 144 (30) | 22 |  |  | 759 | 154 (20) | 47 |  |  |
|  | >30 breaths/min | | 386 | | | 157 (41) | 13 |  |  | 350 | 85 (24) | 44 |  |  |
| Creatinine | | |  | | |  |  | <0.0001 |  |  |  |  | <0.0001 | <0.0001 |
|  | ≤2.0 mg/L | | 615 | | | 186 (30) | 23 |  |  | 773 | 127 (16) | - |  |  |
|  | >2.0 mg/L | | 256 | | | 118 (46) | 12 |  |  | 336 | 112 (33) | 22 |  |  |
| Bilirubin |  | |  | | |  |  | <0.0001 |  |  |  |  | 0.0007 | <0.0001 |
|  | ≤2.0 mg/dL | | 540 | | | 179 (33) | 19 |  |  | 642 | 137 (21) | 46 |  |  |
|  | >2.0 mg/dL | | 164 | | | 85 (52) | 9 |  |  | 185 | 61 (33) | 22 |  |  |
| Thrombocytopenia | | |  | | |  |  | <0.0001 |  |  |  |  | 0.007 | <0.0001 |
|  | >80×10^9^/L | | 714 | | | 225 (32) | 24 |  |  | 991 | 201 (20) | 49 |  |  |
|  | ≤80×10^9^/L | | 152 | | | 78 (51) | 10 |  |  | 119 | 38 (32) | 24 |  |  |
| Albumin |  | |  | | |  |  | 0.246 |  |  |  |  | 0.417 | 0.122 |
|  | ≥25 g/dL | | 284 | | | 99 (35) | 21 |  |  | 376 | 76 (20) | 42 |  |  |
|  | <25 g/dL | | 409 | | | 163 (40) | 12 |  |  | 446 | 121 (27) | 34 |  |  |
| **Predisposing conditions for ARDS** | | | | | | |  |  |  |  |  |  |  |  |
| Sepsis |  | |  | | |  |  | 0.008 |  |  |  |  | 0.446 | 0.094 |
|  | No sepsis | | 117 | | | 27 (23) | 41 |  |  | 23 | 4 (17) | - |  |  |
|  | Sepsis | | 756 | | | 277 (37) | 16 |  |  | 1087 | 235 (22) | 45 |  |  |
| Septic shock | | |  | | |  |  | 0.016 |  |  |  |  | 0.804 | 0.854 |
|  | No septic shock | | 309 | | | 93 (30) | 30 |  |  | 78 | 18 (23) | 43 |  |  |
|  | Septic shock | | 564 | | | 211 (37) | 13 |  |  | 1032 | 221 (21) | 45 |  |  |
| Pneumonia | | |  | | |  |  | 0.273 |  |  |  |  | 0.316 | 0.008 |
|  | No pneumonia | | 249 | | | 81 (33) | 21 |  |  | 566 | 112 (20) | 48 |  |  |
|  | Pneumonia | | 624 | | | 223 (36) | 17 |  |  | 544 | 127 (23) | 41 |  |  |
| Pancreatitis | | | - | | |  |  |  |  | - |  |  |  |  |
|  | No pancreatitis | | | | |  |  |  |  |  |  |  |  |  |
|  | Pancreatitis | | - | | |  |  |  |  | - |  |  |  |  |
| Trauma |  | |  | | |  |  | <0.0001 |  |  |  |  | 0.054 | <0.0001 |
|  | No trauma | | 816 | | | 300 (37) | 16 |  |  | 1091 | 238 (22) | 44 |  |  |
|  | Trauma | | 57 | | | 4 (7) | - |  |  | 19 | 1 (5) | - |  |  |
| Multiple transfusions | | | | | |  |  | 0.978 |  |  |  |  | 0.702 | 0.406 |
|  | No multiple transfusions | | | 798 | | 278 (35) | 18 |  |  | 1071 | 229 (21) | 45 |  |  |
|  | Multiple transfusions | | | 75 | | 26 (35) | 23 |  |  | 39 | 10 (26) | 47 |  |  |
| Aspiration | | | |  | |  |  | 0.119 |  |  |  |  | 0.002 | 0.0005 |
|  | No aspiration | | | 795 | | 273 (34) | 19 |  |  | 1043 | 214 (21) | 48 |  |  |
|  | Aspiration | | | 78 | | 31 (40) | 15 |  |  | 67 | 25 (37) | 16 |  |  |
| Pulmonary injury | | | |  | |  |  | 0.633 |  |  |  |  | 0.317 | 0.059 |
|  | Direct pulmonary injury | | | 208 | | 77 (37) | 16 |  |  | 562 | 111 (20) | 48 |  |  |
|  | External pulmonary injury | | | 665 | | 227 (34) | 18 |  |  | 548 | 128 (23) | 41 |  |  |

ARDS=acute respiratory distress syndrome; APACHE=Acute Physiology and Chronic Health Evaluation; ICU=intensive care unit
