# Supplementary material for: Thrombocytopenia Is Associated with Acute Respiratory Distress Syndrome Mortality: An International Study
Source: PLoS One. 2014 Apr 14;9(4):e94124. doi: 10.1371/journal.pone.0094124 (PMC3986053; doi:10.1371/journal.pone.0094124)
Supplement: Table S2 — Risk factors for ARDS in Boston cohort. (DOCX) [file pone.0094124.s002.docx]

**Supplemental Table S2. Risk factors for ARDS in Boston cohort**

|  | **Non-ARDS** | | **ARDS** | ***p*** |
| --- | --- | --- | --- | --- |
|  | (n =1110) | | (n =873) |  |
| **Female, n (%)** | 450 (41) | | 320 (37) | 0.071 |
| **Age, median (range)** | 66 (55-77) | | 59 (45-72) | <0.0001 |
| **Smoking history, n (%)** |  | |  |  |
| **Baseline severity of illness (1^st^ 24 hours of ICU admission)** | | | | |
| APACHE II, median (range)* | | 23 (20-27) | 24 (21-28) | 0.0004 |
| Systolic BP, <90 mmHg, n (%) | | 833 (75) | 606 (68) | 0.0007 |
| Heart rate, >100 beats/min, n (%) | | 800 (72) | 701 (79) | 0.0002 |
| Respiratory rate, >30 breaths/min, n (%) | | 350 (32) | 388 (44) | <0.0001 |
| Creatinine, >2.0 mg/L, n (%) | | 336 (30) | 266 (30) | 0.881 |
| Bilirubin, >2.0 mg/dL, n (%) | | 185 (22) | 166 (23) | 0.727 |
| Thrombocytopenia, ≤80×10^9^ platelets/L, n (%) | | 119 (11) | 156 (18) | <0.0001 |
| Albumin, <25 g/dL, n (%) | | 446 (54) | 421 (60) | 0.037 |
| **Comorbidities, n (%)** | |  |  |  |
| Diabetes | | 329 (30) | 198 (23) | 0.0003 |
| **Predisposing conditions for ARDS, n (%)** | | |  |  |
| Sepsis syndrome | | 1087 (98) | 768 (86) | <0.0001 |
| Septic shock | | 1032 (93) | 576 (65) | <0.0001 |
| Pneumonia | | 544 (49) | 632 (71) | <0.0001 |
| Pancreatitis | | - | - |  |
| Trauma | | 19 (2) | 59 (7) | <0.0001 |
| Multiple transfusions | | 39 (4) | 77 (9) | <0.0001 |
| Aspiration | | 67 (6) | 78 (9) | 0.019 |
| Direct pulmonary injury vs. external pulmonary injury^†^ | | 548 (49) | 675 (76) | <0.0001 |
| **Clinical outcomes** | |  |  |  |
| 60-Day mortality, n (%) | | 239 (22) | 314 (35) | <0.0001 |

ARDS=acute respiratory distress syndrome; APACHE=Acute Physiology and Chronic Health Evaluation; ICU=intensive care unit

*APACHE II score was calculated with all components within 24 hours of ICU admission;

^†^Pneumonia, aspiration, pulmonary contusions, or sepsis from lower pulmonary source were categorized as direct pulmonary injury; sepsis from an extrapulmonary source, trauma without pulmonary contusions, and multiple transfusions were categorized as external pulmonary lung injury. Patients with both direct and external pulmonary injuries were considered to have direct lung injury.
